# Axillary Needle Biopsy in the Era of American College of Surgeons Oncology Group (ACOSOG) Z0011: Institutional Experience With a Largely Urban Minority Population and Review of the Literature

**DOI:** 10.7759/cureus.24317

**Published:** 2022-04-20

**Authors:** Lydia Choi, Kimberly Ku, Wei Chen, Awni D Shahait, Steve Kim

**Affiliations:** 1 Michael and Marian Ilitch Department of Surgery, Wayne State University School of Medicine, Detroit, USA; 2 Oncology, Illinois Cancer Care, Chicago, USA; 3 Statistics, Wayne State University, Detroit, USA; 4 Oncology, Karmanos Cancer Institute, Detroit, USA; 5 Surgery, Wayne State University Detroit Medical Center, Detroit, USA

**Keywords:** resection, breast cancer, axillary dissection, axillary needle biopsy, axillary ultrasound

## Abstract

Background: The American College of Surgeons Oncology Group (ACOSOG) Z0011 trial demonstrated that sentinel lymph node biopsy (SLNB) alone is adequate for axillary control in patients with one to two positive axillary lymph nodes. However, axillary lymph node dissection (ALND) is required in patients with N1 disease diagnosed with a preoperative needle biopsy. In this report, we determined how many patients could potentially have had SNB alone based on finding only one to two positive nodes in the axilla.

Methods: A retrospective review of patients with positive preoperative axillary needle biopsy undergoing ALND was used to identify rates of high volume axillary disease (>2 positive nodes). Wilcoxon’s rank-sum and Fisher’s exact test were used for statistical analysis. A review of the literature is included for comparison.

Results: 73% of 51 total patients with a positive needle biopsy had >2 positive nodes on axillary dissection. The high-volume axillary disease was significantly more likely with the presence of lymphovascular invasion and extranodal extension.

Conclusions: Patients with positive preoperative axillary needle biopsies have a significantly higher rate of high volume axillary disease. However, at least one-quarter of these patients will have <3 positive nodes and potentially could have been treated with SNB alone.

## Introduction

Sentinel lymph node biopsy (SLNB) has significantly reduced morbidity in the axilla due to breast cancer surgery [1]. Randomized trials have shown that patients with negative sentinel nodes can forgo axillary lymph node dissection (ALND) with fewer complications, the most dreaded of which is lymphedema [2,3]. SLNB, in which only the first echelon lymph nodes draining the breast tumor identified with radioactive and chemical dyes are removed for pathologic analysis, should only be done in patients with clinically negative axilla, and traditionally, this has been defined as no lymph nodes palpable on physical exam. However, this definition may not be clear in the setting of a patient with a difficult axillary physical exam (for instance, those with high BMI) but clearly abnormal imaging findings on preoperative ultrasound. A recent meta-analysis found that ultrasound-guided biopsy assessment of axillary lymph node metastases had a sensitivity of about 80% and a specificity of 98% [4]. In this study, approximately 20% of women with clinically negative axillae were found to have a positive axillary ultrasound-guided biopsy and therefore underwent ALND without initial sentinel lymph node biopsy. This study underscores that a significant proportion of women with a negative physical exam have abnormal axillary lymph node(s) detectable only by ultrasound, leading to the detection of axillary metastasis. For this reason, routine preoperative ultrasound has been advocated to more accurately identify axillary metastasis preoperatively.

However, there is currently no standard protocol for assessing the axilla preoperatively in all patients with breast cancer, and the National Comprehensive Cancer Network (NCCN) recommendation is to consider imaging if needed, while UK guidelines recommend a routine axillary ultrasound, but practices vary widely in the US [5]. The American College of Radiology offers no clear guidelines on when the axilla should be imaged with ultrasound, only recommending that any abnormal lymph node on imaging be biopsied [6].

What is clear is that lymph node metastasis found on preoperative axillary ultrasound-guided biopsy usually changes management, since these patients are treated with complete ALND rather than sentinel node biopsy. However, since the publication of results from the American College of Surgeons Oncology Group (ACOSOG) Z0011 trial, there has been a paradigm shift in the management of the axilla in breast cancer [7-9]. Prior to this trial, standard management in the case of any number of sentinel nodes containing metastasis was to complete ALND. However, Z0011 showed that there was no difference in locoregional recurrence or overall survival at a median of six years with or without axillary dissection when fewer than three sentinel nodes contained metastases in patients with T1 or T2 tumors receiving breast-conserving therapy. In this context, the value of preoperative axillary ultrasound becomes questionable since the finding of a single positive lymph node preoperatively would not necessarily rule out sentinel node biopsy. Indeed, a recent study has shown that 70% of patients with abnormal axillary imaging were found to have fewer than three positive nodes at the time of surgery [10], and the authors concluded that preoperative axillary imaging and biopsy should not be done routinely as it may frequently lead to overtreatment of the axilla. In the current study, we sought to determine if preoperative axillary ultrasound and needle biopsy of axillary nodes were predictive of the need for axillary dissection in our institution, i.e., identifying patients with >2 positive nodes at final pathology.

## Materials and methods

A retrospective chart review was performed after institutional review board approval was obtained. The target population was all female patients with invasive breast cancer undergoing axillary ultrasound and core needle biopsy of a suspicious lymph node from December 1, 2005, to December 1, 2015, at the Barbara Ann Karmanos Comprehensive Cancer Center. The sonographic criteria for selecting suspicious lymph nodes included size, cortical thickening (diffuse or eccentric), loss of fatty hilum, loss of oval shape, and abnormal cortical blood flow. All patients who had axillary dissection or sentinel node biopsy at our institution after a positive core needle biopsy were included in our analysis. The biopsy was performed under US guidance with multiple passes and the adequacy of the biopsy was assessed by pathology. There were 937 evaluable patients. We excluded patients receiving neoadjuvant chemotherapy. A total of 51 patients were included in this analysis.

The total number of positive nodes identified after axillary dissection was used to determine the sensitivity and specificity of preoperative axillary ultrasound for patients who required axillary dissection for more than two positive axillary nodes.

Patient characteristics such as age, tumor size, and the presence of palpable nodes on physical exam were identified. Data were also collected on pathologic tumor size, type, and characteristics such as tumor grade, hormone receptor status, and the presence of lymphovascular invasion.

Descriptive statistics for patient baseline characteristics were summarized within subgroups defined by positive nodal status. Medians with ranges were reported for continuous data, whereas frequencies and percentages were for categorical data. Wilcoxon’s rank-sum test was used for continuous data, and Fisher’s exact test was used for categorical data. All p-values are two-sided with a significance level of 0.05. The results of these analyses should be regarded as exploratory findings, and raw p-values were reported without adjustment for multiple testing. All calculations were performed with R version 3.2.2 (RStudio, Boston, MA).

## Results

A total of 51 patients were found to have a positive axillary needle biopsy before subsequent sentinel node biopsy or axillary dissection. Both lumpectomy (n = 24) and mastectomy (n = 27) patients were included in our analysis. Results are summarized in Table 1.

**Table 1:** Baseline characteristics by the number of positive nodes. The absolute number of patients is indicated for each category along with the range when applicable. Percentages are out of the total number of patients in that column. P-value shows statistical significance only for extranodal extension and lymphovascular invasion.

	<2 positive nodes (n=14)	>2 positive nodes (n=37)	P-value
Median age	55.5 (35,82)	50 (27,83)	0.59
Race			
Caucasian	4 (29%)	12 (32%)	
African American	9 (64%)	21 (57%)	
Other	1 (7%)	4 (11%)	
Median body mass index	28	29	
Imaging			
Ultrasound shows 1 abnormal node	13 (93%)	34 (92%)	
Ultrasound shows multiple abnormal nodes	4 (29%)	10 (27%)	1
Lymph node size >2 cm	7 (50%)	9 (24%)	
Type of surgery			
Sentinel lymph node	3 (21%)	4 (11%)	0.376
Lumpectomy	7 (50%)	17 (46%)	1
Mastectomy	7 (50%)	20 (54%)	
Pathology			
Invasive lobular carcinoma	3 (21%)	3 (8%)	0.514
Invasive ductal carcinoma	11 (79%)	33 (89%)	
Median grade on core biopsy			0.601
1	1 (7%)	5 (14%)	
2	4 (29%)	15 (41%)	
3	4 (29%)	15 (41%)	
Estrogen receptor/progesterone Receptor +	8 (57%)	26 (70%)	
Estrogen receptor/progesterone Receptor −	6 (43%)	11 (30%)	
Human epidermal growth factor receptor 2 +	2 (14%)	8 (22%)	
(Pathologic) T stage			0.757
1	1 (7%)	7 (19%)	
2	12 (86%)	25 (68%)	
3	1 (7%)	4 (11%)	
4	0 (0%)	1 (3%)	
N stage (pathological)			<0.001
1	14 (100%)	8 (22%)	
2	0 (0%)	21 (57%)	
3	0 (0%)	8 (22%)	
Total no. of lymph nodes removed			0.061
Median (min, max)	12.5 (3,24)	17 (7,39)	
No. of positive lymph nodes	1 (1,2)	5 (3,16)	
% with lymphovascular invasion	5 (36%)	28 (76%)	0.019
Extranodal extension	6 (43%)	34 (92%)	0.001
Lymphedema	4 (29%)	10 (27%)	1

About 73% (37) had more than two positive axillary nodes. 27% (n=14) had only one or two positive nodes on axillary dissection and would not have required axillary dissection by Z0011 criteria if all had undergone breast-conserving surgery.

Twenty-four patients underwent breast-conserving surgery. If only patients who underwent breast-conserving surgery were included in the analysis, 29% (n=7) had one or two positive nodes in the axilla and would not have required axillary dissection. While 59% (n=30) of the patients included in this study were African American, 31% (n=16) Caucasian, and 10% (n=5) of other races. Race was not a predictor of requiring axillary dissection.

Forty-four patients had invasive ductal cancer, six patients had invasive lobular cancer, and one patient had mixed ductal and lobular breast cancer. Three out of six patients with lobular cancer had more than two positive nodes in the axilla. Only five patients underwent sentinel node biopsy before axillary dissection. Two additional patients underwent sentinel node biopsy only. Three patients had <3 positive nodes, two of which were ones who only had sentinel node biopsy done.

Receptor status

About 67% (n=34) had ER/PR+ tumors and 20% (n=10) had H2N+ tumors. Of 37 patients with more than two positive nodes, 70% (n=26) had ER/PR+ tumors and 22% (n=8) had H2N+ (one equivocal) tumors.

Palpable nodes or multiple abnormal axillary nodes by ultrasound

Fifty-seven percent (n=29) had palpable nodes on physical exam. Twenty-seven percent (n=14) of all patients were identified to have multiple abnormal lymph nodes in the axilla preoperatively by ultrasound. There was overlap in 10 patients who had both palpable nodes and multiple abnormal nodes on ultrasound. Of patients with multiple abnormal nodes on ultrasound alone, 71% (n=10) had more than two positive nodes in the axilla. When both palpable nodes and abnormal ultrasound were evaluated together, 6 out of 10 patients had more than two positive nodes.

Lymph node morphology or size

Thirty one percent (n=16) of patients had lymph nodes greater than 2 cm in the widest dimension of pathology. Of these patients, 11 (69%) had an extranodal extension. However, there was no statistical correlation between lymph node size and the presence of extranodal extension (p = 0.2884).

Tumor size and presence of lymphovascular invasion

The median T stage of all patients in this study was 2. Of patients who would have required axillary dissection by Z0011 criteria, the median T stage was 2.

Lymphovascular invasion was present in 65% of patients who would require axillary dissection by Z0011 criteria. The microscopic extranodal extension was present in the lymph nodes of 78% of patients. Both of these factors were significantly associated with high volumes of axillary disease.

## Discussion

Before ACOSOG Z0011, axillary ultrasound and needle biopsy had clear benefits. Knowledge of axillary metastasis before surgery allowed more accurate staging, which resulted in the direction of patients to neoadjuvant chemotherapy or to axillary dissection at the time of surgery. Unnecessary sentinel node biopsies were avoided. However, now in the era post-ACOSOG Z0011, the benefit of axillary ultrasound is less well defined.

One advantage is that it usually leads to the identification of a group of patients with high volume axillary disease. The majority of patients in our study (73%) were found to have more than two positive nodes on axillary dissection. Boland et al. reported that 74% of patients with positive axillary needle biopsies have more than two positive nodes on axillary dissection [11]. Verheuvel et al. also found similar numbers, with 70% of patients having more than two positive nodes on axillary dissection if they were diagnosed by preoperative needle biopsy [12]. Among all the reviewed articles, the median number of positive nodes, when reported, was at least 3 [13] (Table 2).

**Table 2:** Summary of other studies examining patients with positive preoperative axillary needle biopsies. The majority of patients in these studies have 50% or more patients with high volume axillary disease. Predictive factors are not consistently identified. US: ultrasound; LN: lymph node.

Study	n (number with positive preoperative axillary needle biopsy)	>2 positive nodes (%)	Predictors for high volume axillary disease (if available)	Median # positive nodes (if available)
Yang et al. [14]	346	61%	>2 abnormal nodes on US	
Liang et al. [15]	202	58%		5
Liu et al. [16]	79	61%	>1 abnormal node on US	
Lim et al. [17]	175	60%	>2 abnormal nodes on US, cortical thickness > 4 mm	
Verheuvel et al. [12]	302	70%	Tumor size, lymphovascular invasion, palpable nodes, ER- status	
Boland et al. [11]	439	86%		3
Dihge et al. [13]	24	54% (N2 or N3 disease)	Size of nodal metastases	4
Zhu et al. [18]	118	60%	Nodal cortical thickness > 3.5 mm	
Kramer et al. [19]	191	58%	Multifocality, tumor diameter, progesterone receptor	
Boone et al. [20]	199	50%		
Farrell et al. [21]	169			5
Caudle et al. [7]	190	48%		4
Hieken et al. [22]	82	51%	LN size, lymphovascular invasion, number of sonographically suspicious LN	5
Van Wely et al. [23]	157	50% (N2 or N3)		

This is in contrast to sentinel node biopsy positive patients, who mostly have one or two positive nodes [24]. The two groups have different risk profiles. When data from the randomized trials of sentinel node biopsy versus axillary dissection are also compared to our data, the majority of patients (approximately 65-70%) in the randomized trials only had one or two positive nodes, whereas the majority of patients in our study had three or more [3,8,25]. Our data reinforce the evidence that axillary metastasis identified by preoperative needle biopsy is more frequently associated with high volume axillary disease.

However, routine use of axillary ultrasound on every cancer diagnosis results in more frequent identification of nodal metastasis. Pilewskie et al. recommend against routine axillary ultrasound [10]. Their study found that 70% of abnormal ultrasounds, when followed up with sentinel node biopsy only, resulted in incorrect identification of patients who require axillary dissection based on the total number of positive nodes.

The methods of performing axillary ultrasound, the criteria for needle biopsy of lymph nodes, and the type of surgery performed after needle biopsy (sentinel node biopsy vs. axillary dissection) affect the positive predictive value of axillary ultrasound. It is problematic to compare the number of positive nodes from a group of patients undergoing sentinel node biopsy to a group undergoing axillary node dissection. Data from ACOSOG Z0011 showed that additional positive non-sentinel nodes were found in 27% of patients who had positive sentinel node biopsy and went on to have axillary dissection [8]. Therefore, we can assume that additional axillary metastases would be found in a similar fraction of patients who undergo sentinel node biopsy only and have node-positive disease. Furthermore, patients who have axillary dissection, in comparison, may be "overdiagnosed" with the disease as a result of more careful searching. This can explain, in part, the significant difference in the number of positive nodes found in our study compared to sentinel node biopsy-only studies.

Several studies, including ours, have sought to identify predictive markers for high volume axillary disease at presentation, which would accurately direct patients to axillary biopsy and dissection or sentinel node biopsy.

Our study shows that patients with an abnormal ultrasound and a positive preoperative axillary needle biopsy are very likely to have high volume axillary disease. Previous studies have clearly shown worse outcomes for African American women with breast cancer, and a higher volume of axillary disease at presentation correlates with this finding [19,26].

However, race was not a predictor for high volume axillary disease in our study. This may be the result of the method of comparison and the small sample size [27]. The majority of the patients included in our study have a high disease burden. A comparison within this group did not show that race was a predictor for more metastatic nodes. However, the majority of the patients included in the study were African American (60%), which is double the average African American population treated for breast cancer overall at our institution (30%).

Predictive factors for high volume disease in other studies included multiple abnormal nodes identified by axillary ultrasound or palpable nodes on physical exam (Table 2). In our study, when palpable nodes were used as a predictor for more than two positive nodes, 72% (n=21) of 29 patients with palpable nodes had more than two positive nodes. This is concordant with findings in other studies, where clinically palpable nodes were found to be malignant on pathologic analysis 59-82% of the time [28]. Nodal size, a proxy for palpable nodes, was found to be predictive of high volume axillary disease in articles by Dihge and Hieken [13,22,29].

Hieken et al. also showed a correlation between multiple abnormal nodes on ultrasound and high volume axillary disease [22]. If multiple abnormal nodes were seen on axillary ultrasound, twice as many of these patients (32%) were noted to have more than two positive nodes on final pathology, as compared to 15% of those with only one abnormal node on ultrasound. Of patients in our study who had both physical exam and ultrasound findings suggestive of high volume axillary disease, 60% had more than two positive axillary nodes.

## Conclusions

Our results suggest that patients with positive axillary needle biopsies are very likely to have high volume axillary disease. The small sample size may limit the ability to detect predictive factors. However, our study shows that 27% of patients with a positive preoperative needle biopsy will have two or fewer nodes positive and hence may be able to avoid the morbidity of axillary lymphadenectomy.

Finally, the majority of patients at our institution who had positive preoperative axillary needle biopsies received neoadjuvant chemotherapy. This may indicate the future direction of axillary management. If axillary ultrasound and biopsy confirm the presence of metastasis, these patients will undergo neoadjuvant chemotherapy and subsequent sentinel node biopsy instead of immediate axillary dissection. If there is adequate down-staging, this may result in avoiding axillary lymphadenectomy even in those patients with initial advanced regional metastatic disease.
